# Effects of Antibiotics on the Intestinal Microbiota of Mice

**DOI:** 10.3390/antibiotics9040191

**Published:** 2020-04-17

**Authors:** Frederik Boetius Hertz, Andries E. Budding, Malieka van der Lugt-Degen, Paul H. Savelkoul, Anders Løbner-Olesen, Niels Frimodt-Møller

**Affiliations:** 1 Department of Clinical Microbiology, Hvidovre University Hospital, 2650 Copenhagen, Denmark; 2 Department of Clinical Microbiology, Slagelse Hospital, 4200 Slagelse, Denmark; 3 Department of Medical Microbiology & Infection Control, University Medical Centers, Location VUmc, 1081HV Amsterdam, The Netherlands; Dries.Budding@inbiome.com (A.E.B.); maliekadegen@gmail.com (M.v.d.L.-D.); paul.savelkoul@mumc.nl (P.H.S.); 4 In Biome, Science Park, 116, 1081XG Amsterdam, The Netherlands; 5 Department of Medical Microbiology, NUTRIM School of Nutrition and Translational Research in Metabolism, Maastricht University medical Center, 6202 AZ Maastricht, The Netherlands; 6 Department of Biology, University of Copenhagen, 2200 Copenhagen, Denmark; lobner@bio.ku.dk; 7 Department of Clinical Microbiology, Rigshospitalet, 2100 Copenhagen, Denmark; Niels.Frimodt-Moeller@regionh.dk

**Keywords:** intestinal microbiota, 16S, IS-pro, antibiotics, dicloxacillin

## Abstract

Studies on human and mouse gastrointestinal microbiota have correlated the composition of the microbiota to a variety of diseases, as well as proved it vital to prevent colonization with resistant bacteria, a phenomenon known as colonization resistance. Antibiotics dramatically modify the gut community and there are examples of how antibiotic usage lead to colonization with resistant bacteria [e.g., dicloxacillin usage selecting for ESBL-producing *E. coli* carriage], as shown by Hertz et al. Here, we investigated the impact of five antibiotics [cefotaxime, cefuroxime, dicloxacillin, clindamycin, and ciprofloxacin] on the intestinal microbiota in mice. Five different antibiotics were each given to groups of five mice. The intestinal microbiotas were profiled by use of the IS-pro analysis; a 16S–23S rDNA interspace [IS]-region-based profiling method. For the mice receiving dicloxacillin and clindamycin, we observed dramatic shifts in dominating phyla from day 1 to day 5. Of note, diversity increased, but overall bacterial load decreased. For ciprofloxacin, cefotaxime, and cefuroxime there were few overall changes. We speculate that antibiotics with efficacy against the abundant anaerobes in the gut, particularly Bacteroidetes, can in fact be selected for resistant bacteria, disregarding the spectrum of activity.

## 1. Introduction

Medical care is increasingly impacted by colonization and infection of patients by antibiotic resistant bacteria [1,2]. Furthermore, studies on human and mouse gastrointestinal microbiota have correlated the composition of the microbiota to a variety of diseases, as well as proved it vital to prevent enteric infections and possibly colonization with pathogenic, resistant bacteria [1,2,3,4]. The latter is due to a phenomenon known as colonization resistance [1,2,3,4]. As such, it is well accepted that the microbiota plays an essential role in maintaining human health [1,2,3,4].

In addition to the diversity within one individual’s microbiota (alpha diversity), there is great diversity in the microbial composition between individuals (beta diversity) [2]. Yet, most of the microbiotas are dominated by just four phyla—the Bacteroidetes, Firmicutes, Actinobacteria, and Proteobacteria [2]. Firmicutes and Bacteroidetes generally account for more than 90% of the bacterial population in the colon [2]. One of the most dramatic modifications to the gut community is that caused by antibiotic treatment, but the impact of antibiotics on the gastrointestinal microbiota is not fully understood [1,5]. Antibiotic treatment can cause selection of drug-resistant bacteria and subsequent colonization or infection with multidrug-resistant bacteria, generally regarded as inevitable collateral damage [6]. Patients receiving antibiotic treatment may develop a dysbiotic gut microbiota, characterized by a shift in dominating phyla, reduced diversity, and intestinal overgrowth of multi-drug resistant or opportunistic pathogens, e.g., *Escherichia coli* and *Enterococcus* spp. [3,4,7,8]. Colonization with resistant bacteria causes a risk of infection, especially in patients with co-morbidities [1,2]. 

The process of colonization of the gut by antibiotic resistant bacteria following the administration of antibiotics, has been shown in mice where antibiotics administered subcutaneously induced colonization by orally administered ESBL-producing *E. coli* (EPE) or vancomycin-resistant Enterococci (VRE) [4,9]. Even dicloxacillin, which is considered an antibiotic with a narrow-spectrum activity towards mainly Gram-positive bacteria, was found to select EPE in a mouse intestinal colonization model [9,10]. To gain a better understanding of the impact of different antibiotics on gut microbiota, we investigated the impact of five antibiotics (cefotaxime, cefuroxime, dicloxacillin, clindamycin, and ciprofloxacin) on the intestinal microbiota in mice, by use of the IS-pro analysis; a 16S–23S rDNA interspace (IS)-region-based profiling method. 

## 2. Results

**Figure 1:**
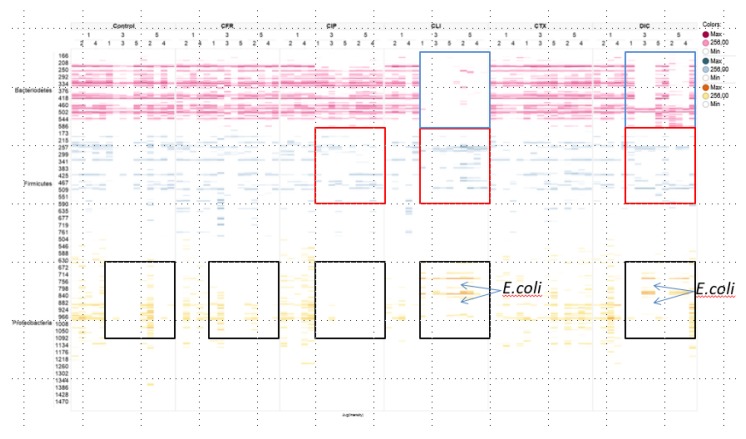
Bacterial phylum profiles per mouse and antibiotic group (five mice per antibiotic or control), established by 16S rRNA analyses. Colour intensity of the bands indicates quantities of respective species. Each band represents a microbial interspace (ITS) fragment, corresponding to an operational taxonomy unit (OTU). Fragments are sorted into their corresponding phylum. No bands; indicate the absence of bacterial species. **“1” =** Day 1, **“3”** = Day 3, and **“5”** = Day 5. Numbers below indicate that data are shown for each individual mouse. Five mice in two different cages. **Blue boxes** indicate the shift in Bacteroidetes composition for clindamycin and dicloxacillin. **Red boxes** indicate the shift in Firmicutes composition for ciprofloxacin, clindamycin, and dicloxacillin. **Black boxes** show depletion of Proteobacteria in most antibiotics groups (and in controls). We have indicated the concurrent invasion of *E. coli* in clindamycin and dicloxacillin treated mice. **Control**: No antibiotics given; **CFR**: Cefuroxime; **CIP**: Ciprofloxacin; **CLI**: Clindamycin; **CTX**: Cefotaxime; **DIC**: Dicloxacillin.

**Figure 2:**
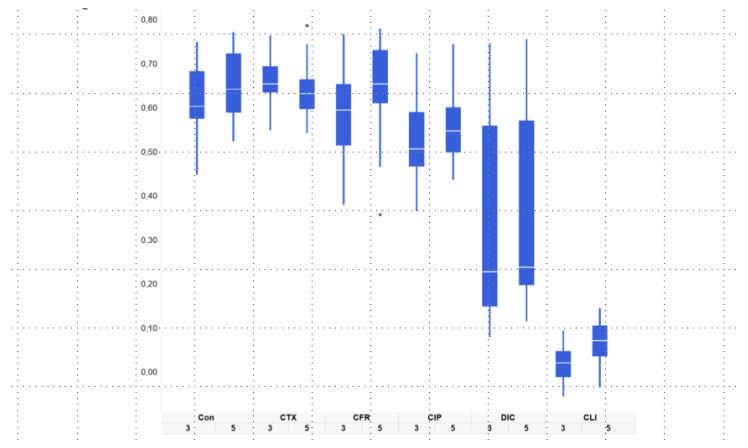
Box plot of the similarity of gut microbiota at day 3 and 5 compared to the initial microbiota. The y-axis represents cosine similarity values, for pairs of IS-profiles of the microbiota at different dates (higher value means higher similarity to pretreatment microbiota). Shown here is the normal variation and the variation induced by antibiotic treatment. Boxes represent the cosine similarity values for all five mice in each group.

**Figure 3:**
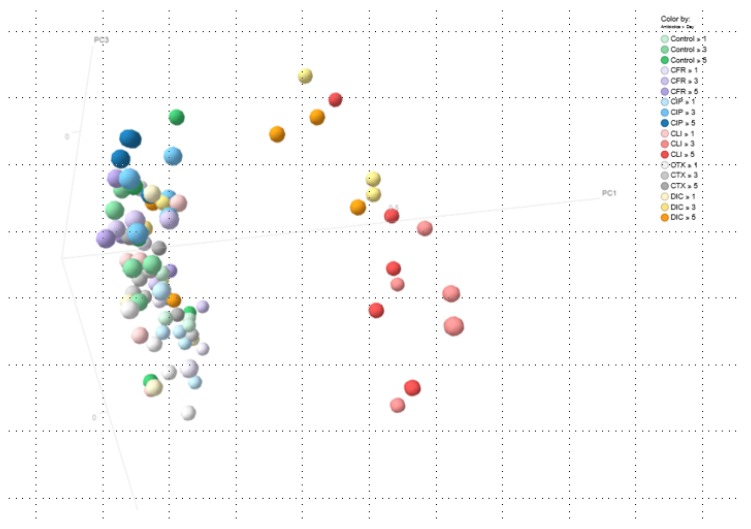
The principal coordinates analysis (PCoA) based on cosine distance between samples from all mice on all days. Each dot represents a gut microbiota profile. Colours show the antibiotic group and day. The further dots are separated, the larger the dissimilarity between associated gut microbiota profiles. The group to the right (**red** and **orange**) represent individual profiles from mice in the clindamycin and dicloxacillin groups at days 3 and 5. These are clearly separated from the rest of the samples. PCoA starts by putting the first point at the origin, and the second along the first axis (e.g., PC1). Each axis can represent a phylum or bacterial species. Further points can be added, which usually means adding a second axis followed by a possible third axis and so forth (e.g., PC2 and PC3 etc.). A successful PCoA will, therefore, generate a 2–3 axes. Each object has a ‘score’ along each axis. The combined scores delivering the position in the plot. Via PCoA, we visualize the dissimilarities, or distances, between samples from mice receiving antibiotics. Interpretation of a PCoA plot is straightforward: Objects closer to one another are more similar than those further away. The points illustrate the normal variation for controls and the variation induced by antibiotic treatment. Antibiotic treatments were given once a day for three days (day 1 to day 3). The faeces was collected on day 1 (prior to the antibiotic treatment), on day 3, and finally on day 5 (two days after the end of treatment), respectively. **“1”** = Day 1, **“2”** = Day 2, and **“5”** = Day 5. **Control**: No antibiotics given; **CFR**: Cefuroxime; **CIP**: Ciprofloxacin; **CLI**: Clindamycin; **CTX**: Cefotaxime; **DIC**: Dicloxacillin.

Results are based either on beta-diversity (mentioned as beta-similarity) between total microbiota profiles or on the individual species level, stratified per main phylum. The main phyla were Bacteroidetes (Gram-negative anaerobic, including the *Bacteroides* spp.), Firmicutes (most Gram-positives including *Clostridia* spp., *Enterococcus* spp., and *Staphylococcus* spp.) and Proteobacteria (including Enterobacterales such as *Escherichia coli* as well as *Pseudomonas* spp.). Results are presented as profiles per mouse and antibiotic group (Figure 1, Figure 2 and Figure 3) or as pooled data for all five mice in each group (Figure 2). The resulting p-values for similarity are shown in Table 1. The alpha diversity (Shannon index) is illustrated in Supplementary Figure S1 and p-values are shown in Supplementary Table S1. Of note, Supplementary Figure S1, shows the alpha diversity for Bacteroidetes, Firmicutes, and Proteobacteria, respectively. Supplementary Table S1 shows data for all phyla combined.

In brief, antibiotic treatments were given subcutaneously in the neck once a day for three consecutive days: The first treatment was administered on day 1, the second dose on day 2, and third and final dose on day 3 [9]. The faeces was collected on day 1 (prior to the antibiotic treatment), on day 3, and finally on day 5 (two days after the end of treatment), respectively.

Figure 1 illustrates the bacterial profiles per mouse and antibiotic group, established by 16S rRNA analyses, on the bacterial species level (shown as individual bars) and sorted per phylum (colours) to demonstrate the influence of antibiotic treatment on the entire microbiota. The five antibiotics used here had different effects on the microbiota as a whole and dissimilar effects on the different bacterial phyla. Clindamycin and dicloxacillin had a large impact on the Bacteroidetes community. In the clindamycin exposed group, Bacteroidetes from all five mice were affected. In the dicloxacillin group, Bacteroidetes from three of five mice were severely affected, while the impact in the remaining two mice was less outspoken. Interestingly, some Bacteroidetes species were unaffected in all mice receiving dicloxacillin. The impact on species within the phylum Firmicutes was heterogenous. In none of the mice did we see the elimination of the entire phylum. However, clindamycin, dicloxacillin, and ciprofloxacin appeared to inhibit some genera in the Firmicutes, while others proliferated. In the phylum, Proteobacteria, a lot of variation was observed between different days, in all groups, including the control group. Of note, in the clindamycin and dicloxacillin groups, the proliferation of *E. coli* was seen (Figure 1). In contrast, ciprofloxacin eliminated all species from the Proteobacteria phylum in four out of five mice on day 3, and in all mice on day 5 (Figure 1). The two cephalosporins, cefotaxime and cefuroxime, had the most limited effects on the murine gut microbiota. An impact on Proteobacteria was seen in the cephalosporin groups, but these variations were similar to those observed among mice in the control group. Furthermore, the cephalosporins had almost no effect on Bacteroidetes, the phylum dominated by anaerobic bacteria, nor on Firmicutes (Figure 1).

Figure 2 shows the cosine similarity, as a measure of similarity, of gut microbiota at day 3 and 5 compared to the initial (pretreatment) microbiota composition. Hence, Figure 2 depicts the normal variation for the control group, as well as the variation induced by antibiotic treatment. 

We observed a large variation in the effects of treatment with different antibiotics on the microbiota (Figure 2). For mice receiving dicloxacillin and clindamycin, the gut microbiota was impacted most markedly after treatment, as can be seen by the low cosine correlations and wide intervals in the box plot of Figure 2. 

Finally, the principal coordinates analysis (PCoA) was used to visualize the similarity of all gut microbiota profiles of mice from all groups on all days (Figure 3). Here, the most outspoken dissimilarities, or distances, among samples were seen in the groups receiving dicloxacillin and clindamycin, on day 3 and 5. Figure 1, Figure 2 and Figure 3 show that clindamycin and dicloxacillin induced major shifts among all phyla, shifts that were sustained throughout the study. For ciprofloxacin, cefotaxime, and cefuroxime we observed a high similarity between pre- and post-treatment microbiota (Figure 2; Figure 3).

The beta similarity between samples were calculated by a cosine correlation. The microbiota similarity for all timepoints and all mice within an antibiotic group was calculated. Resulting similarity scores were compared for timepoint 1 vs. 3 and 3 vs. 5 for each antibiotic group against the same values from the control group. A two-tailed t-test was used to calculate the p-values for R between Control T1–T3 and antibiotic T1–T3 and R between Control T1–T5 and antibiotic T1–T5.

## 3. Discussion

The background for this study was our previous observation in the intestinal colonization model in mice where we compared the ability of the five antibiotics used in the present study (cefotaxime, cefuroxime, dicloxacillin, clindamycin, and ciprofloxacin) to select for an orally administered EPE during and after treatment with the antibiotics administered for three days only [9]. While the two cephalosporins were selected for EPE as predicted, the two narrow-spectrum antibiotics dicloxacillin and clindamycin to our surprise were selected for EPE, equally well. The choice of dicloxacillin as the standard antibiotic for staphylococcal infections in Denmark, has been based on its excellent effect in these infections and on the assumption that it is a narrow-spectrum antibiotic with minor effect on the normal flora. While the impact of clindamycin and ciprofloxacin treatment on the mouse gut microbiome has been studied in detail before, usually in context with medical or probiotic interventions, we have found no studies on the effect on the mouse gut microbiome of cefuroxime or dicloxacillin (or any other isoxazoyl penicillin (oxa-, cloxa-, flucloxa-, or nafcillin)). There are studies focusing on the mouse gut microbiome using other cephalosporins or clindamycin and studies focusing on the human gut microbiome using cephalosporins and ciprofloxacin [11,12,13]. Most of these studies have focused on susceptibility to infection, yet to our knowledge there are no studies investigating the effect of dicloxacillin on the gut microbiome nor have any study compared the effect with other antibiotics [11,12,13]. Finally, the majority of studies have focused on susceptibility to infection with specific pathogens such as *Clostridium difficile, enterococci spp., salmonella spp.,* EPE, etc. and not the overall impact on the microbiota [9,11,12,14,15].

In this study, we found that the five antibiotics had very different effects on the different phyla in the murine gut microbiota. We observed that dicloxacillin and clindamycin had a dramatic impact on Bacteroidetes with possibly secondary proliferative effects on Proteobacteria. Inversely, ciprofloxacin reduced Proteobacteria to below the detection limit with minor effects on the remaining phyla, while the cephalosporins had limited and transitory effects on all phyla. Figure 1 highlights the vast differences between effects of antibiotics on the phyla, while Figure 2 and Figure 3 show how treatment induces a shift in the composition of the microbiota, as a whole. While this 16S rRNA-based study revealed which phyla and genera were present and how they were affected, this study did not include the aim of metagenomic sequencing to describe the presence of specific genes or sub-divide species [16].

The broad antibacterial effect on anaerobes is well known for clindamycin, explaining the decrease in Bacteroidetes during treatment. It is, however, expected that dicloxacillin has an inadequate effect on anaerobic Gram-negatives, but has shown some activity against the anaerobic Gram-positive bacteria, i.e., various *Streptococci* spp. and *Clostridia* spp. [17,18]. This could explain the decrease in Firmicutes seen here. The susceptibility of different genera belonging to Bacteroidetes towards dicloxacillin may vary depending on production of beta-lactamases, but whether Bacteroidetes are susceptible or the impact here is derived effect is not clear [17]. Furthermore, species of the Bacteroidetes phylum very often encode beta-lactamases related to cephalosporinase [19]. It has been shown, in vitro, that when these beta-lactamases are carried by outer membrane vesicles (OMVs), they help protect other species in the environment, as these surface-associated beta-lactamases could degrade cephalosporins nearby [19]. This could potentially be the phenomenon we see in mice receiving cephalosporins, with a minimal impact on Bacteroidetes and other phyla, irrespective of time (Figure 1) [17,19]. Yet, we would then expect surface-associated beta-lactamases to protect the microbiota against dicloxacillin, as well. 

The broad spectrum of activity of clindamycin, including Bacteroidetes, explains the pronounced effect on the gut microbiota, with a marked shift in Proteobacteria towards dominant colonization with *E. coli* and a shift in Firmicutes species. This clearly illustrates a potential for colonization with ESBL-producing *E. coli* (EPE) and vancomycin-resistant *Enterococci* (VRE). Furthermore, clindamycin has been associated with *Clostridium difficile* enterocolitis in patients. As *C. difficile* also belong to Firmicutes, our results demonstrate a potential high risk for collateral damage during antibiotic treatment, which could have a severe consequence for patients receiving clindamycin treatment [8]. Largely, clindamycin overturns the microbiota, changing the composition and even though the diversity of the microbiota is still high for some phyla, there are fewer bacteria overall (Supplementary data 1).

We demonstrate that dicloxacillin has the potential to select for EPE (Figure 1). This has previously been shown in a mouse model but was not reproduced in a case-control study of urinary tract infection (UTI) caused by EPE. However, a few patients only received dicloxacillin [9,20]. Dicloxacillin seems to inhibit various species of the gut microbiota, creating a niche for opportunistic colonizers such as *E. coli.* Yet, we lack evidence to firmly claim that any changes in Proteobacteria are caused by changes in other phyla. Nonetheless, we observe that dicloxacillin has the ability to inhibit the gut microbiota, providing a potential risk of colonization with EPE. Surprisingly, dicloxacillin inhibited most Bacteroidetes in three out of five mice, indicating a potential for profound impact on the microbiota and considerable collateral damage. Dicloxacillin (similar to oxacillin, cloxacillin, or flucloxacillin) is often prescribed for skin and soft-tissue infections, with a treatment duration of ≥2 weeks. Thus, this could lead to a profound alteration of the composition of gut microbiota and allow for colonization of opportunistic pathogens [21] which would be an obvious subject of a clinical study. In this current study, all antibiotics were administered for three consecutive days only. A longer period of administration and subsequent comparison of results between “short” and “long” treatment duration could have provided more evidence for the impact on the microbiota. This may well be the scope of succeeding studies, especially since previous studies for ciprofloxacin showed that the changes persisted for several weeks with the extent of restoration to the baseline composition of the microbiome as highly subject-dependent [11].

The high diversity of the microbiome has been associated with a healthy gut and a reduced risk of colonization with VRE, ESBL, as well as a low risk of *C. difficile* infection [3,4]. Yet, we see here an impact on the gut microbiota, creating a microbiota which contains fewer bacteria overall (Supplementary data 1). Hence, it is not the diversity of the microbiota only, but also the total load of bacteria and likely the contributions of specific bacterial species, which are specifically related to colonization resistance [1,2]. 

The compositions of the intestinal microflora of laboratory mice have been examined by Krych et al. to evaluate the similarities to the flora of the human gut [22]. There were some quantitative differences, but mouse and human feces, to a large extent, had similar representatives of phyla and a substantial segment of common genera. Hence, lab mice and humans share the same basic bacterial species in the gut [22].

Colonization studies of the mouse gut by bacteria have been used in a range of mouse models, with many similarities between methods [23,24]. It has previously been shown, that a human-simulated dosing of antibiotics gives the most constant levels of the drug in mice—who have a higher elimination rate [25]. Yet, a single daily subcutaneous dose can produce similar levels of drugs in mouse feces, to those seen in humans [26]. This dosing frequency of antibiotic, nonetheless, does not precisely mimic the exposures seen in patients [26]. Therefore, all mouse dosages were calculated based on human doses (in mg per kg of body weight) from our own pharmacokinetic (PK) studies or from previously published mouse studies [26,27,28,29,30,31,32,33]. This study has several limitations. We did not investigate changes in the levels of different proteins, short-chain fatty acids, or possible anti-microbial molecules produced by the commensal microbiota [2,4]. Furthermore, mice are coprophagic which could alter phyla distribution among mice living together in the same cages and could lead to rapid recolonization of the gut after antibiotic administration. Thus, coprophagy may explain why we see some mice with complete eradication of the microbiota and some with apparently intact microbiota.

We did not measure the antibiotic concentration in the faeces. Thus, any day-to-day changes or mouse-to-mouse differences in the faecal antibiotic concentration are unknown. Finally, we do observe some variability among mice receiving the same antibiotics, as illustrated by mice receiving dicloxacillin and Bacteroidetes data, indicating some intra-group unpredictability. 

## 4. Materials and Methods 

### 4.1. Mouse Model

We used a mouse model based on a mouse intestinal colonization model, as previously described [9]. The present study was carried out at identical settings as the preceding study, according to current guidelines and with outbred NMRI mice (Harlan, the Netherlands) [9]. In this current study, we used five antibiotics, each given to five mice (cefotaxime, cefuroxime, clindamycin, dicloxacillin, and ciprofloxacin). All mouse-doses were chosen to mimic the serum antibiotic concentrations achieved in humans, as previously described (Table 2) [9]. Antibiotics were administered subcutaneously once a day for three consecutive days. We included a control group consisting of five mice who received saline only. This group was used as the baseline for natural variability of the microbiota. 

The use of animals was approved by the Danish Centre for Animal Welfare and treatments given subcutaneously in the neck once a day for three consecutive days (day 1 to day 3) as doses of 0.25 mL [9]. The faeces was collected on specific preselected days of the study: Day 1 (prior to the antibiotic treatment), day 3, and day 5 (two days after the end of treatment), respectively. The faeces collection was performed directly from each mouse during bowel movements. 

Table 2. Antibiotics used for treatment. All doses were administered subcutaneously once a day for three consecutive days. Doses were calculated from an expected average weight of mice (weight given by the provider), except for clindamycin, which was administered as in a previously published study [8]. We included a control group consisting of five mice who did not receive antibiotics. This group was used as the baseline for natural variability of the microbiota.

### 4.2. IS-Profiling of the Intestinal Microbiome

The intestinal microbiome was profiled by an analysis of faeces samples by use of IS-pro, introduced by Budding et al. in 2010 [34]. IS-pro is a clinically validated gut microbiota analysis tool based on the species-specific length polymorphism of the 16S-23S interspace (IS) region and phylum-specific sequence polymorphisms of 16S rDNA. All samples were analyzed with phylum-specific fluorescently labelled primers. Resulting IS profiles consist of a set of peaks with: (A) A specific length, measured in nucleotides reflecting lengths of IS fragments and (B) a specific height, measured in relative fluorescence units, thus reflecting the quantity of the PCR product and a specific fluorescent label, corresponding to the bacterial phylum. Quantitative results can, therefore, be given per species, per phylum group, or per operational taxonomic unit (OTU), corresponding to individual peaks. In this present study, specific optimizations were made for murine gut microbiota. DNA isolation, amplification, and capillary gel electrophoresis were performed as described by Budding et al. 2010 [34]. All data optimization and analysis were performed by IS Diagnostics Ltd. with the in-house developed software in combination with the Spotfire software package (Tibco). We used a Student’s t-test to compare the quantitative 16S PCR analysis between mice [7]. Diversity was calculated with unrarefied data, as rarefication is not relevant to the IS-pro data. For alpha diversity the Shannon index was used, for beta diversity both the cosine correlation (nonphylogenetic) and principal coordinate analysis (nonphylogenetic) were used.

### 4.3. Principal Coordinates Analysis (PCoA)

The principal coordinates analysis (PCoA) is a method to explore and visualize similarities or dissimilarities among samples from a different time-point [35]. PCoA starts by putting the first point at the origin, and the second along the first axis (e.g., PC1). Each point represents a single sample and each axis can represent a phylum or bacterial species. Further points can be added, which usually means adding a second axis followed by a possible third axis, etc. (e.g., PC2 and PC3) [35]. A successful PCoA will, therefore, generate a 2–3 axes. Here, we show the two first principal coordinates (PC1 and PC2). Each object has a ‘score’ along each axis. The combined scores delivering the position in the plot. Via PCoA, we visualize the dissimilarities, or distances, between samples from mice receiving antibiotics [35]. Interpretation of a PCoA plot is straightforward: Points that are closer together represent microbial communities that are more similar in composition and the distance between points represent how compositionally different the samples are [35]. 

### 4.4. Statistical Analyses

The alpha diversity was calculated per mouse, per timepoint, and per antibiotic as the Shannon diversity index. A two-tailed paired t-test was used to calculate the p-values for alpha diversity between T1 and T3 and for diversity between T1 and T5.

The beta similarity between samples were calculated by the cosine correlation. The microbiota similarity for all timepoints and all mice within an antibiotic group was calculated. Resulting similarity scores were compared for timepoint 1 vs. 3 and 3 vs. 5 for each antibiotic group against the same values from the control group. A two-tailed t-test was used to calculate the p-values for R between Control T1–T3 and antibiotic T1–T3 and R between Control T1–T5 and antibiotic T1–T5. Of note, the gut microbiota counts per OTU cannot be normally distributed, based on the data sparsity and zero inflation. However, Shannon diversity is a transformation of the data which reduces these data to a range of values. These values do show a normal distribution and a t-test can be performed. Similarly, we do not directly assess beta-diversity. Instead, we are comparing measured sets of beta-diversity measurements (beta-diversity over timepoints in the control set vs. beta diversity over time for each antibiotic). These measures are also a transformation of the data that follow a normal distribution. Here too, a t-test is warranted. 

## 5. Conclusions

We can conclude that different antibiotics alter the microbiota very differently, and the impact on different phyla varies markedly. We speculate that antibiotics with a presumed narrow-spectrum of activity, such as dicloxacillin, can in fact be selected for EPE and overall cause substantial collateral damage as seen with broad-spectrum antibiotics [1,2]. 

We find a need for more detailed investigations to clarify antibiotic susceptibility of common gut commensals and investigate the long-term impact of antibiotics and the risk for proliferation of pathogens such as EPE, VRE, and *C. difficile.*

## Figures and Tables

**Table 1:** P-values for the beta similarity between day 1 (T1) and day 3 (T3), as well as day 1 and day 5 (T5).

Groups Receiving Antibiotics vs. Controls	T1–T3	T1–T5
**CFR**	0.47194285	0.79228912
**CIP**	0.00086195	7.16 × 10^−5^
**CLI**	5.12 × 10^−35^	1.72 × 10^−34^
**CTX**	0.00590382	0.43879666
**DIC**	1.45 × 10^−6^	9.86 × 10^−8^

**Table 2:** Antibiotics used for treatment.

Antibiotic	Dose Human	Cmax, Human µg/mL	Needed Dose Per Mice mg/kg	Cmax, Mouse µg/mL	Dose Given. Calculated by Weight Per Mouse in mg/day
**Cefuroxime**	**1.5 g iv**	**65**	**120**	**50–60**	**4**
**Cefotaxime**	**1 g iv**	**40**	**60**	**100**	**2**
**Dicloxacillin**	**1 g iv**	**30–40**	**60**	**90**	**2**
**Clindamycin**	**1.8 g iv**	**6**	**36**	**8**	**1.4**
**Ciprofloxacin**	**0.4 g iv**	**4**	**15**	**2**	**0.5**

## Supplementary Materials

The following are available online at https://www.mdpi.com/2079-6382/9/4/191/s1, Figure S1: Box plot of the alpha diversity of gut microbiota at day 3 and 5 compared to the initial microbiota, Table S1: The alpha diversity was calculated per mouse, per timepoint, per antibiotic as the Shannon diversity index.
